# Editorial: Climate change, human health, and health systems

**DOI:** 10.3389/frhs.2025.1615206

**Published:** 2025-08-15

**Authors:** Munyaradzi Saruchera, Morenike Oluwatoyin Folayan, Bethuel S. Ngcamu, Walter Musakwa, Margaret Kaseje, Godfred O. Boateng

**Affiliations:** ^1^ Africa Centre for Inclusive Health Management, Stellenbosch University, Cape Town, South Africa; ^2^ Department of Child Dental Health, Obafemi Awolowo University, Ile-Ife, Nigeria; ^3^ Public Administration and Management, University of South Africa, Pretoria, South Africa; ^4^ Geography, Environmental Studies & Energy, University of Johannesburg, Johannesburg, South Africa; ^5^ Health Science, Tropical Institute of Community Health and Development, Kisumu, Kenya; ^6^ School of Global Health, York University, Toronto, ON, Canada

**Keywords:** climate, health, systems, disasters, environment, energy

**Editorial on the Research Topic**
Climate change, human health, and health systems

## Introduction

Climate change is impacting human life in many ways and affecting health systems and outcomes of healthcare services. This calls for a multidisciplinary approach that includes health policies, environmental science and economics. Research presented here explored the impact of climate change and implications on health outcomes and included actionable strategies for mitigation and adaptation.

The research on pricing strategy and pharmaceutical supply chains (Lu et al.) highlights the important role that pricing strategies play in optimizing pharmaceutical supply chains. Amid climate change challenges, there will be need for health systems to adapt pricing frameworks that reflect environmental costs and promote sustainable practices while ensuring access to essential medications.

A study conducted in Hong Kong on the association between ambient temperature and hospital stay length (Long et al.) revealed that increased ambient temperatures correlated with extended hospital stays for patients suffering from cardiopulmonary diseases. This finding emphasizes the importance of proactive health system adjustments to manage the heat-related health impacts exacerbated by climate change and implies the need for improved services, hospital infrastructure and capacity planning.

The research on Sustainable Solarized Vaccine Cold Chain System in Lebanon (Kapuria et al.) showcases the implementation of a solarpowered cold chain system for vaccines, illustrating a practical step towards sustainable healthcare. By transitioning to environmentally viable energy sources, health systems can improve vaccine distribution efficiency while reducing carbon footprints, aligning with global health sustainability goals.

The study on Impact of Infectious Disease Experience on Household Consumption in Rural China (Han et al.) illustrates how infectious disease outbreaks influences household consumption behaviors. Understanding these dynamics will assist in addressing the economic ramifications of healthcare service crises, including climate change disasters and their negative effects on communities.

The research on One Health Economics (Leandri et al.) argues that economics must integrate with health science to tackle pressing public health challenges. By adopting a One Health perspective, health systems can better address the complex interactions between climate, ecosystems, and human health, leading to more effective interventions.

The article on Climate Crisis and HIV Prevention (Williams et al.) explores the relationship between climate change and HIV transmission patterns. It provides actionable recommendations for HIV prevention strategies through the lens of climate adaptation, particularly enhancing PrEP programs to cater to populations vulnerable to both climate and HIV risks.

The research on Climate Change Distress and Impairment in Germany (Konig et al.) points to the need for health systems and services to incorporate mental health into climate change mitigation strategies, recognizing that psychological well-being is integral to overall public health.

## Broader context and implications

The convergence of climate change and human health presents significant challenges for health systems globally (1). As climates shift and health risks escalate, there is a need for adaptive measures that go beyond the traditional health systems frameworks. The findings from these studies present ways of adapting health systems to address these challenges that includes economic adaption, infrastructure and capacity building, a One Health framework and inclusion of mental health. An economic strategy that re-evaluates pricing, funding, and resource allocation in health systems may mitigate the effects of climate change. Integrating sustainable practices can not only protect health but also enhance economic viability (2). Health systems need to invest in infrastructure that can withstand climate-related pressures and include expanding hospital capacities and developing emergency response protocols to address heat-related health issues.

Emphasizing the One Health framework encourages collaboration between various sectors, including agriculture, environmental science, and health. Interdisciplinary approaches are needed for effective strategies to combat climate impacts on health. Incorporating mental health services within climate adaptation frameworks will likely improve community resilience and overall health outcomes. The exploration of HIV transmission dynamics amid changing climates points to the need to integrate climate considerations into preventive healthcare programs to reduce vulnerability among at-risk populations.

However, Africa's exclusion is striking, given the impacts of the climate crisis. Inclusive and equitable global solutions to the climate crisis must place Africa and other regions' realities, needs, and innovations at the forefront of a climate-proofed approach to health management for all. Holistic and integrated research to support practice and policies that promote and protect wellbeing and human health while simultaneously improving ecosystem restoration and climate change mitigation globally are needed. This requires change to break down the silos in research, policymaking, and institutional arrangements, and enabling cross-sectoral, holistic and interdisciplinary approaches and solutions.

## Conclusion

Climate change is adversely impacting health infrastructure, services and workforce. The intersection of climate change and health requires a multidisciplinary approach to address the complex and multiple interrelated issues. The above studies' findings provide for the development of interdisciplinary, targeted and innovative strategies and solutions for climate-proofed health services and facilities, policy development, sustainable resource planning and management in the context of climate change. In view of the synthesis, a multiplicity of the specific interdisciplinary priorities for research needs to be explored which include risk assessment and adaptation, extreme events and air pollution, sustainable development, infectious disease and phenology, atmospheric sciences, public health, environment sciences, and policy development both in the Global South and North. Furthermore, an integrated health system should be explored which is focusing on systematic impact of climate change with an aim to achieve effective and efficient risk governance. Another interdisciplinary approach should focus on heath system resilience to explore adaptative and transformative strategies to determine the relationships between climate change, migration, and health systems.

Specific interdisciplinary areas for research include improvement of health impact assessment and implementation; development of relevant technologies, infrastructures, policies and human resources; and promotion of research on transformational change towards sustainability. The One Health approach is interdisciplinary and provides a broad research agenda that can benefit health policymaking, resource planning and management. By leveraging the findings of current research, health systems can be better equipped to navigate the evolving landscape of healthcare service and interdisciplinary research collaboration leads to new discoveries and a meaningful difference in health services. A public and private partnerships should be recommended as they have a potential to create a comprehensive and complementary data infrastructure which is accessible to health services and policy research throughout the country. In addition, to reform the health care system, both the public and the private sector should invest on a cost-effective data infrastructure to monitor the impact of health care related and climate change reform initiatives. It is suggested that research funders should strategically invest to quality systematic literature reviews aiming to inform health care management and policy-makers to support them to effectively evaluate the local adaptation processes.

Future researchers should affirm that quality systematic literature reviews effectively inform health care management and policy-making ensuring that managers and policy-makers understand and make rationale decision informed by the findings emerged from the synthesis.
